# Comparison of erector spinae plane block with paravertebral block for thoracoscopic surgery: a meta-analysis of randomized controlled trials

**DOI:** 10.1186/s13019-023-02343-w

**Published:** 2023-10-27

**Authors:** Jinghua Pang, Jiawen You, Yong Chen, Chengjun Song

**Affiliations:** Department of Cardiothoracic Surgery, Fenghua District People’s Hospital of Ningbo, Zhejiang, China

**Keywords:** Erector spinae plane block, Paravertebral block, Thoracoscopic surgery, Pain scores, Randomized controlled trials, Meta-analysis

## Abstract

**Introduction:**

The efficacy of erector spinae plane block versus paravertebral block for thoracoscopic surgery remains controversial. We conduct a systematic review and meta-analysis to explore the impact of erector spinae plane block versus paravertebral block on thoracoscopic surgery.

**Methods:**

We have searched PubMed, EMbase, Web of science, EBSCO, and Cochrane library databases through March 2022 for randomized controlled trials (RCTs) assessing the effect of erector spinae plane block versus paravertebral block on thoracoscopic surgery. This meta-analysis is performed using the random-effect model.

**Results:**

Seven RCTs are included in the meta-analysis. Overall, compared with erector spinae plane block for thoracoscopic surgery, paravertebral block results in significantly reduced pain scores at 12 h (SMD = 1.12; 95% CI 0.42 to 1.81; *P* = 0.002) and postoperative anesthesia consumption (SMD = 1.27; 95% CI 0.30 to 2.23; *P* = 0.01), but these two groups have similar pain scores at 1-2 h (SMD = 1.01; 95% CI − 0.13 to 2.15; *P* 0.08) and 4–6 h (SMD = 0.33; 95% CI − 0.16 to 0.81; *P* = 0.19), as well as incidence of nausea and vomiting (OR 0.93; 95% CI 0.38 to 2.29; *P* = 0.88).

**Conclusions:**

Paravertebral block may be better for the pain relief after thoracoscopic surgery than erector spinae plane block.

## Introduction

Thoracoscopic surgery is a less invasive and traumatic surgical procedure for both minor and major oncological lung surgeries, and it is able to improve post‑operative respiratory function and reduce hospital length of stay [1–3]. Thoracoscopic surgery has been widely used to treat various diseases such as esophageal cancer and lung cancer [4–6]. However, 25% of patients are estimated to experience moderate‑to‑severe pain after thoracoscopic surgery [7]. Inadequate analgesia delays patient recovery and prolongs the hospital stays.

Due to the limited efficacy and adverse events of current analgesic methods, many kinds of regional anesthesia techniques such as thoracic epidural analgesia and paravertebral block have been developed to alleviate post‑operative pain after thoracoscopic surgery [8, 9]. Erector spinae plane block also obtains widespread application because of simple application and safety [10]. However, the optimal regional anaesthesia technique among erector spinae plane block versus paravertebral block is not well established for thoracoscopic surgery [10–14]. This meta-analysis aims to investigate the efficacy and safety of erector spinae plane block versus paravertebral block for thoracoscopic surgery.

## Materials and methods

Ethical approval and patient consent are not required because this is a systematic review and meta-analysis of previously published studies. The systematic review and meta-analysis are conducted and reported in adherence to PRISMA (Preferred Reporting Items for Systematic Reviews and Meta-Analyses) [15, 16].

### Search strategy and study selection

Two investigators have independently searched the following databases (inception to March 2022): PubMed, EMbase, Web of science, EBSCO, and Cochrane library databases. The electronic search strategy is conducted using the following keywords: “erector spinae plane block” OR “ESPB” AND versus “paravertebral block” OR “PVB” AND “thoracoscopic” OR “thoracoscopy”. We also check the reference lists of the screened full-text studies to identify other potentially eligible trials.

The inclusive selection criteria are as follows: (1) population: patients undergoing thoracoscopic surgery; (2) intervention: erector spinae plane block; (3) comparison: paravertebral block; (4) study design: RCT. We exclude patients with spinal deformities, infection at or near the puncture site, abnormal coagulation, a history of allergy to local anesthetics, a history of psychiatric disorders or inability to cooperate.

### Data extraction and outcome measures

We have extracted the following information: author, number of patients, age, male, body mass index, American Society of Anesthesiologists (ASA, I/II) and detail methods in each group etc. Data have been extracted independently by two investigators, and discrepancies are resolved by consensus. We also contact the corresponding author to obtain the data when necessary. The primary outcomes are pain scores at 1–2 h, 4–6 h and 12 h. Secondary outcomes include postoperative anesthesia consumption, nausea, and vomiting. Pain scores were evaluated by visual analogue score (VAS).

### Quality assessment in individual studies

Methodological quality of the included studies is independently evaluated using the modified Jadad scale [16, 17]. There are 3 items for Jadad scale: randomization (0–2 points), blinding (0–2 points), dropouts and withdrawals (0–1 points). The score of Jadad Scale varies from 0 to 5 points. An article with Jadad score ≤ 2 is considered to be of low quality. If the Jadad score ≥ 3, the study is thought to be of high quality [18].

### Statistical analysis

We estimate the mean difference (MD) or standard mean difference (SMD) with 95% confidence interval (CI) for continuous outcomes and odd ratio (OR) with 95%CI for dichotomous outcomes. The random-effect model is used when encountering significant heterogeneity, otherwise fixed-effect model is applied. Heterogeneity is reported using the I^2^ statistic, and I^2^ > 50% indicates significant heterogeneity [19]. Whenever significant heterogeneity is present, we search for potential sources of heterogeneity via omitting one study in turn for the meta-analysis or performing subgroup analysis. Publication bias is not evaluated because of the limited number (< 10) of included studies. All statistical analyses are performed using Review Manager Version 5.3 (The Cochrane Collaboration, Software Update, Oxford, UK).

### Quality of evidence

The quality of evidence for each outcome was evaluated based on the methodological quality and the confidence in the results, and it was assessed by GRADE recommendations as high quality, moderate quality, low quality, or very low quality [20].

## Results

### Literature search, study characteristics and quality assessment

**Fig. 1:**
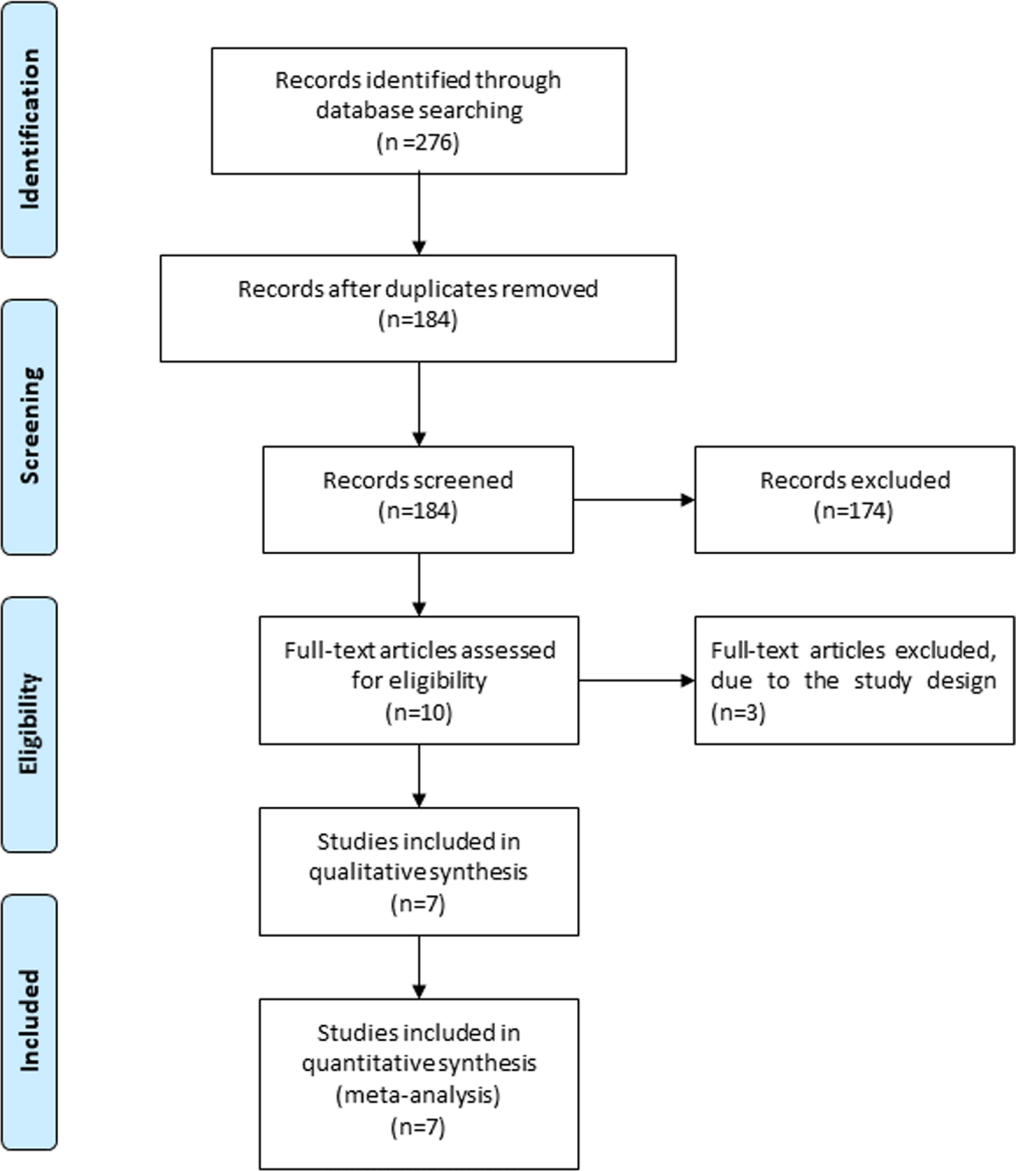
Flow diagram of study searching and selection process

A detailed flowchart of the search and selection results is shown in Fig. 1. 276 potentially relevant articles are identified initially. 92 duplicates and 174 papers after checking the titles/abstracts were excluded. Three studies were removed because of the study design and seven RCTs were ultimately included in the meta-analysis [10–14, 21, 22].

The baseline characteristics of the seven eligible RCTs in the meta-analysis are summarized in Table 1. The seven studies are published between 2019 and 2022, and total sample size is 411. Erector spinae plane block and paravertebral nerve block were performed by using bupivacaine or ropivacaine. Among the seven studies included here, three studies report pain scores at 1–2 h, 4–6 h and 12 h [10, 12, 22], six studies report postoperative anesthesia consumption [10–14, 21], as well as four studies report nausea and vomiting [10, 11, 14, 22]. Jadad scores of the seven included studies vary from 4 to 5, and all seven studies are considered to have high quality according to quality assessment (Table 2).

**Table 1:** Characteristics of included studies

NO	Author	ESPB group						PVB group						Operation	Analgesic medication	Outcomes	Jada scores
		Number	Age (years)	Male (n)	Body mass index (kg/m^2^)	ASA (I/II)	Methods	Number	Age (years)	Male (n)	Body mass index (kg/m^2^)	ASA (I/II)	Methods				
1	Zhang 2022	22	54.41 ± 7.61	11	25.56 ± 3.01	7/15	ESPB with 30 ml of 0.5% ropivacaine hydrochloride	22	54.32 ± 6.56	10	25.47 ± 2.65	9/13	PVB with 30 ml of 0.5% ropivacaine hydrochloride	Elective thoracoscopic pulmonary lobectomy	0.05 mg/kg of midazolam, 0.5 μg/kg of sufentanil, 0.6 mg/kg of rocuronium, and 0.3 mg/kg of etomidate Were given sequentially by intravenous infusion	Pain scores at 1–2 h, 4–6 h and 12 h, postoperative analgesic consumption, nausea and vomiting	4
2	Fu 2022	20	57.25 ± 11.25	14	23.4 ± 2.46	12/8	ESPB with 20 ml of 0.5% ropivacaine	22	58.63 ± 6.04	8	23.79 ± 2.78	14/8	PVB with 20 ml of 0.5% ropivacaine	Video-assisted thoracoscopic surgery	Propofol 2.0 mg/kg, sufentanil 0.4 µg/kg and cis‑atracurium 0.2 mg/kg	Postoperative analgesic consumption, nausea and vomiting	4
3	Turhan 2021	35	53.31 ± 9.03	19	24.38 ± 1.57	13/19	ESPB with 20 mL of 0.5% bupivacaine	35	53.97 ± 7.34	16	23.78 ± 2.04	13/18	PVB with 20 mL of 0.5% bupivacaine	Thoracoscopic lung surgery	Propofol (2 mg/kg), fentanyl (3 mcg/kg) and rocuronium (0.5 mg/kg)	Pain scores at 1–2 h, 4–6 h and 12 h, postoperative analgesic consumption	4
4	Zhao 2020	33	59 ± 5	18	–	11/21	ESPB with 15 mL of 0.4% ropivacaine	33	57 ± 6	11	–	9/24	PVB with 15 mL of 0.4% ropivacaine	Video-assisted thoracic surgery	Etomidate (0.1 mg/kg), propofol (1 mg/kg), sufentanil (0.3 μg/kg) and cis-atracuronium (0.15 mg/kg)	Postoperative analgesic consumption	4
5	Çiftçi 2020	30	47.33 ± 10.21	15	–	16/14	ESPB with 20 mL of 0.25% bupivacaine	30	47.53 ± 10.43	15	–	11/19	PVB with 20 mL of 0.25% bupivacaine	Video assisted thoracic surgery	Propofol (2–2.5 mg/kg), fentanyl (1–1.5 µg/kg), and rocuronium bromide (0.6 mg/kg)	Postoperative analgesic consumption, nausea and vomiting	5
6	Chen 2020	24	51.6 ± 10.4	13	22.9 ± 2.6	9/15	ESPB with 6.7 ml of 0.375% ropivacaine	24	58.1 ± 7.0	15	23.5 ± 2.4	9/15	PVB with 7 ml of 0.375% ropivacaine	Elective thoracoscopic partial pulmonary resection surgery	Sufentanil 0.5 μg/kg, propofol 1.5–2.0 mg/kg and rocuronium 0.8 mg/kg	Postoperative analgesic consumption	5
7	Taketa 2019	41	70 ± 7	23	23.6 ± 3.4	2/29	ESPB with 20 mL of 0.2% levobupivacaine	40	67 ± 8	25	23.4 ± 3.2	2/29	PVB with 20 mL of 0.2% levobupivacaine	Video- assisted thoracic surgery	Fentanyl (50 µg), remifentanil (0.2–0.5 µg/kg/min) and rocuronium (0.6–1 mg/kg)	Pain scores at 1–2 h, 4–6 h and 12 h, nausea and vomiting	4

ASA: American Society of Anesthesiologists

**Table 2:**
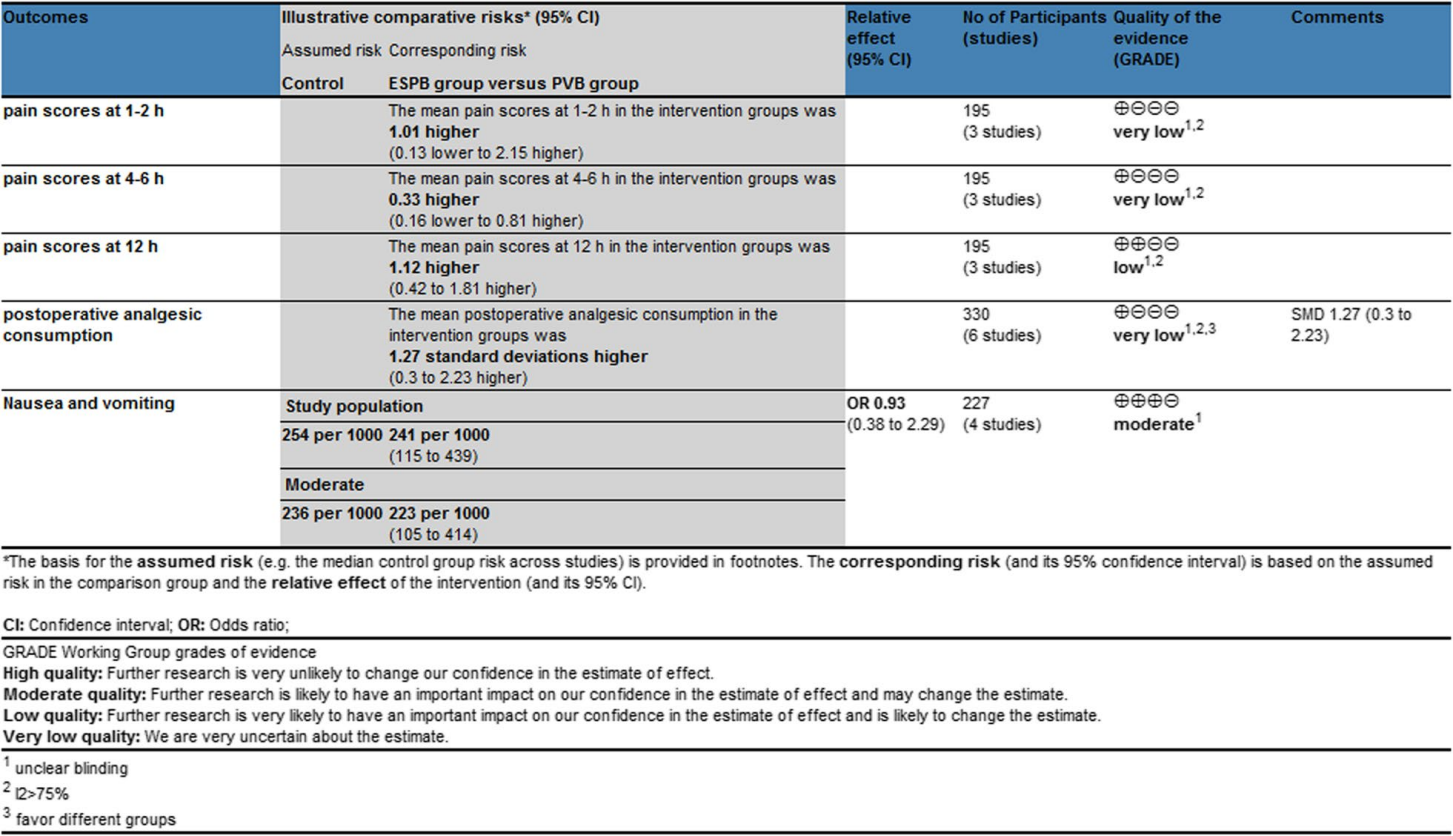
The quality of evidence for each outcome by GRADE recommendations

### Primary outcomes: pain scores at 1–2 h, 4–6 h and 12 h

**Fig. 2:**
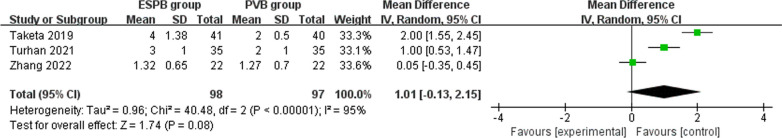
Forest plot for the meta-analysis of pain scores at 1–2 h

**Fig. 3:**
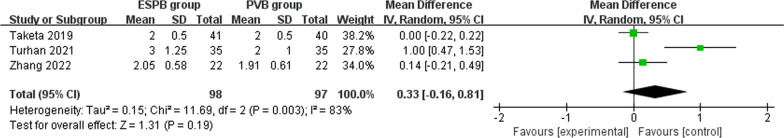
Forest plot for the meta-analysis of pain scores at 4–6 h

**Fig. 4:**
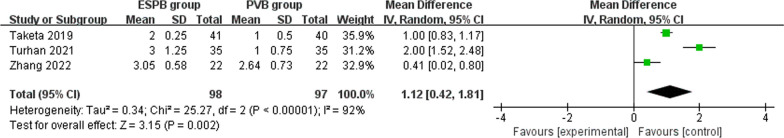
Forest plot for the meta-analysis of pain scores at 12 h

**Fig. 5:**
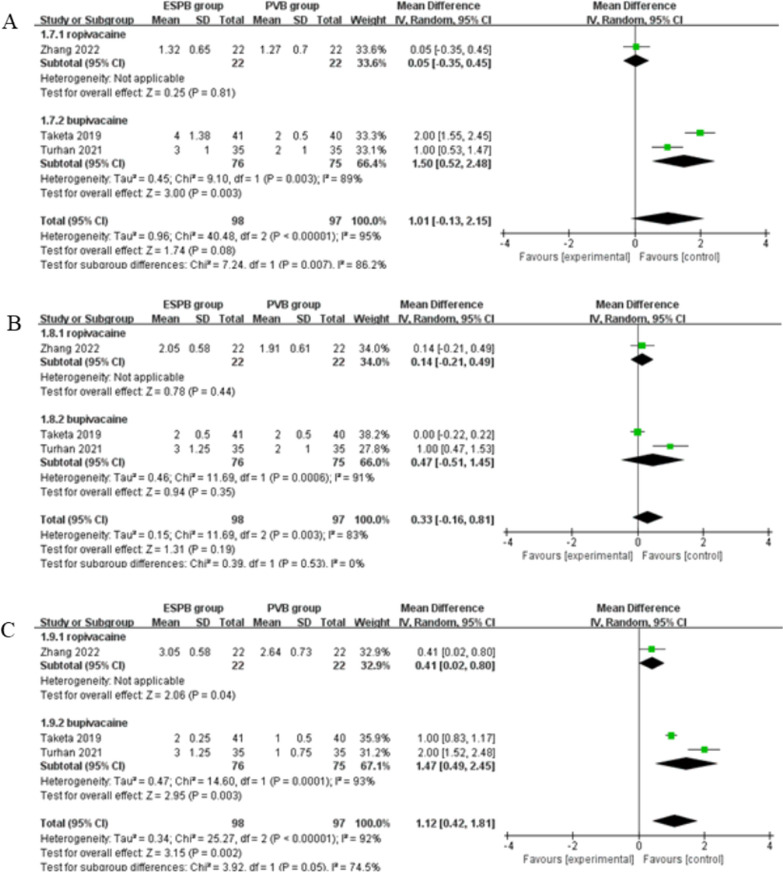
Forest plot for the subgroup analysis of pain scores at **A** 1–2 h, **B** 4–6 h and **C** 12 h

**Fig. 6:**
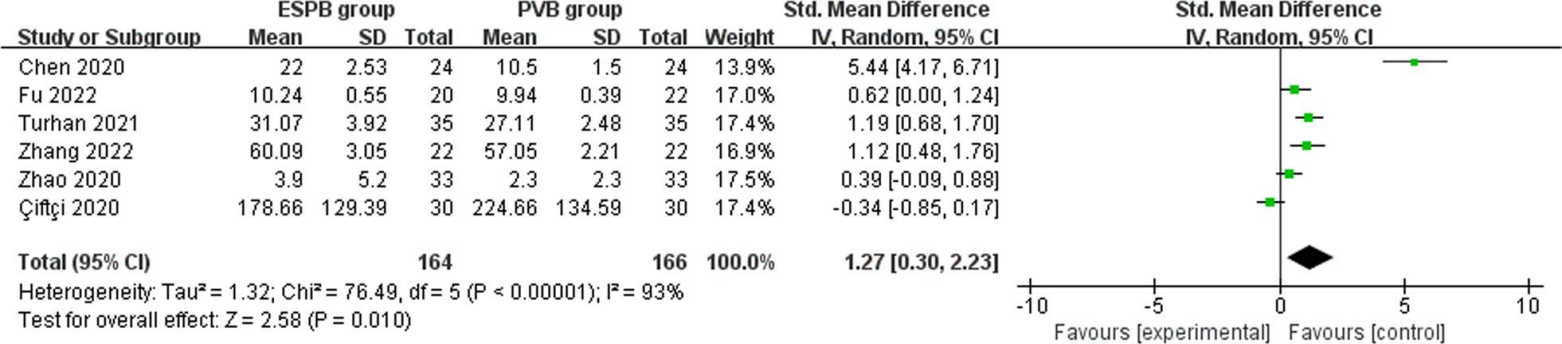
Forest plot for the meta-analysis of postoperative analgesic consumption

The results suggested that compared to paravertebral block for thoracoscopic surgery, erector spinae plane block results in similar pain scores at 1–2 h (very low quality, SMD = 1.01; 95% CI − 0.13 to 2.15; *P* = 0.08) with significant heterogeneity among the studies (I^2^ = 95%, heterogeneity *P* < 0.00001, Fig. 2) and 4–6 h (very low quality, SMD = 0.33; 95% CI − 0.16 to 0.81; *P* = 0.19) with significant heterogeneity among the studies (I^2^ = 83%, heterogeneity *P* = 0.003, Fig. 3), but is associated with significantly higher pain scores at 12 h (low quality, SMD = 1.12; 95% CI 0.42 to 1.81; *P* = 0.002) with significant heterogeneity among the studies (I^2^ = 92%, heterogeneity *P* < 0.00001, Fig. 4).

### Sensitivity analysis

Significant heterogeneity is only observed among the included studies for primary outcomes, but there is still significant heterogeneity when performing sensitivity analysis via omitting one study in turn or subgroup analysis based on anesthetic drugs to detect the heterogeneity (Fig. 5).

### Secondary outcomes

**Fig. 7:**
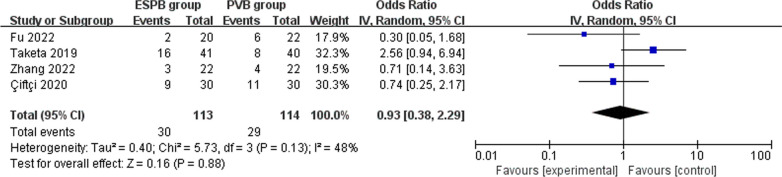
Forest plot for the meta-analysis of nausea and vomiting

Erector spinae plane block needs increased postoperative anesthesia consumption (very low quality, SMD = 1.27; 95% CI 0.30 to 2.23; *P* = 0.01; Fig. 6) than paravertebral block for thoracoscopic surgery, but the incidence of nausea and vomiting is comparable between erector spinae plane block and paravertebral block (moderate quality, OR 0.93; 95% CI 0.38 to 2.29; *P* = 0.88; Fig. 7).

## Discussion

Our meta-analysis included seven RCTs and 411 patients. The results suggested that paravertebral block led to substantially reduced pain scores at 12 h and postoperative anesthesia consumption than erector spinae plane block for thoracoscopic surgery, but pain scores at 1–2 h and 4–6 h were similar between two groups.

Many patients still suffer from obvious postoperative pain after thoracoscopic surgery, and needs pharmacologic and regional interventions [23–28]. Multimodal analgesia methods has been widely developed and include nonsteroidal anti-inflammatory drugs, opioids, patient-controlled analgesia (PCA), infiltration analgesia and thoracal epidural block [29]. There are many nerve block methods that are developed for thoracoscopic surgery. Thoracic epidural analgesia (TEA) is a commonly used method for analgesia following thoracotomy, but results in high risk of complications [30]. Both erector spinae plane block and paravertebral nerve block demonstrate important potential in managing postoperative pain for thoracoscopic surgery [10, 11, 31, 32].

Regarding the sensitivity analysis, significant heterogeneity is seen when performing the analysis by omitting one study in turn or subgroup analysis based on anesthetic drugs. It may be caused by several factors including different analgesic drugs (i.e. ropivacaine and bupivacaine) and various concentrations (e.g. ropivacaine 0.25% and 0.5%). In addition, the detail methods and procedures of thoracoscopic surgery are different due to various diseases, and may produce different baseline pain intensity.

Our results found that paravertebral block showed significantly better analgesic efficacy than erector spinae plane block for thoracoscopic surgery. The possible reasons are speculated as, paravertebral block is a nerve block technique by which local anesthetic is injected directly into the thoracic paravertebral space to block the thoracic spinal nerve and the branches as well as the sympathetic trunk, and the local anesthetic could spread cranially and caudally through the loose connective tissue of the thoracic paravertebral space [33], as well as laterally to the intercostal and epidural spaces [34]. These can provide analgesia comparable to that of the thoracic segmental epidural block [11].

In addition, paravertebral block and erector spinae plane block demonstrated similar incidence of nausea and vomiting in our meta-analysis. There were no adverse events such as pneumothorax, nerve injury or local hematoma [10]. This meta-analysis has several potential limitations. Firstly, our analysis is based on only seven RCTs, and more RCTs with larger sample size should be conducted to explore this issue. Next, different types, concentrations, and methods of anesthetic drugs in included RCTs may have an influence on the pooling results. Finally, different thoracoscopic surgeries produce various baseline pain intensity.

## Conclusions

Paravertebral block may be superior to erector spinae plane block for pain control after thoracoscopic surgery.

## Data Availability

Not applicable.

## Abbreviations

RCTs: Randomized controlled trials
MDs: Mean differences
CIs: Confidence intervals
RRs: Risk ratios
